# Case for diagnosis. Radiotherapy-induced pemphigus vegetans^☆^

**DOI:** 10.1016/j.abd.2021.09.005

**Published:** 2022-03-14

**Authors:** Hiram Larangeira de Almeida Jr, Antônia Larangeira de Almeida, Pedro Henrique Evangelista Martinez, Ana Letícia Boff

**Affiliations:** a Pós-Graduação em Saúde e Comportamento, Universidade Católica de Pelotas, Departamento de Dermatologia, Universidade Federal de Pelotas, Pelotas, RS, Brazil; b Liga de Dermatologia, Universidade Federal de Pelotas, Pelotas, RS, Brazil; c Laboratório Dermapat and Santa Casa de Misericórdia de Porto Alegre, Porto Alegre, RS, Brazil

**Keywords:** Radiotherapy, Pemphigus vegetans

## Abstract

A 73-year-old male patient developed a poorly differentiated squamous cell carcinoma in the anal canal nine months ago. He was treated with two cycles of 5-fluorouracil and cisplatin and concomitant radiotherapy (6 MeV linear photon accelerator, total dose of 54 Gy), with complete remission. Since forty-five days he presentes a painful perianal and intergluteal erosion with circinate pustular borders. Light microscopy showed pseudoepitheliomatous hyperplasia of the epidermis with microabscesses of inflammatory cells (neutrophils and eosinophils) and acantholytic keratinocytes . Indirect immunofluorescence was positive for IgG, with an intercellular pattern, 1:80 titer. The diagnosis of radiotherapy-induced pemphigus vegetans was established and there was significant regression with oral prednisone (40 mg) and topical betamethasone.

## Case report

**Figure 1:**
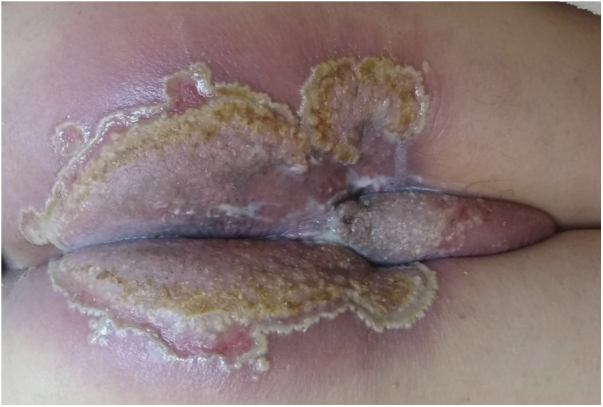
Initial clinical aspect with perianal and intergluteal erosion with circinate pustular edges and some isolated pustules.

**Figure 2:**
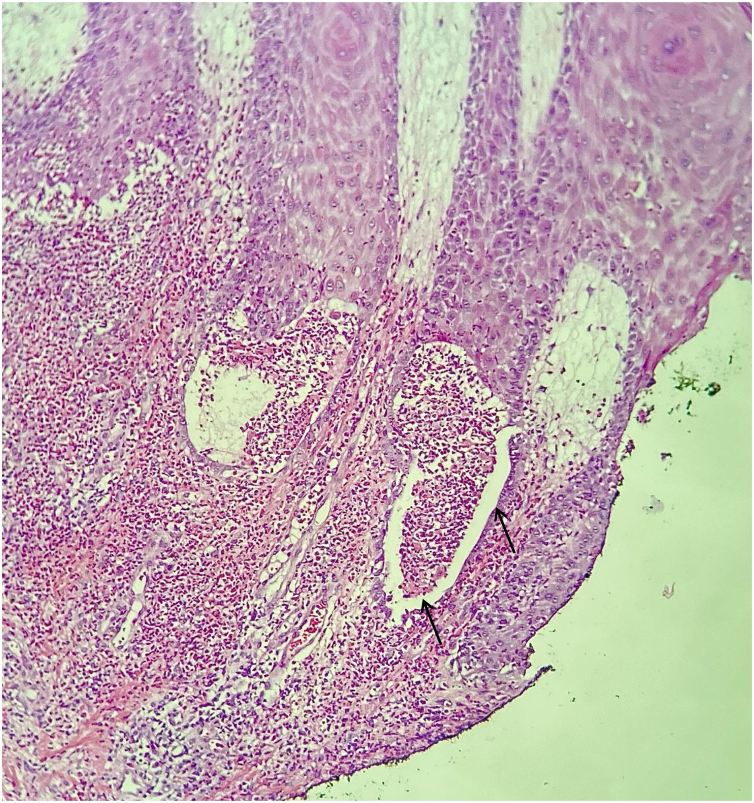
Light microscopy showing clefts and polymorphonuclear microabscess (Hematoxylin & eosin, ×150).

**Figure 3:**
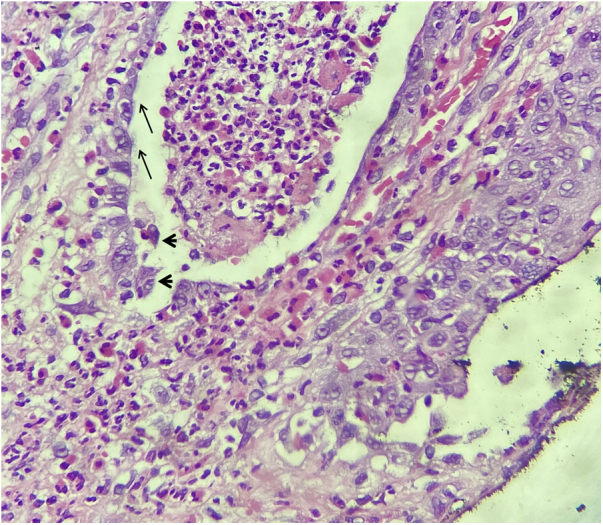
Light microscopy detailing the clefts and polymorphonuclear microabscess with suprabasal cleavage (arrows) and acantholytic keratinocytes (arrowheads); (Hematoxylin & eosin, ×400).

A 73-year-old male patient developed a vegetating lesion in the anal canal nine months ago; the histopathology showed a non-keratinizing poorly differentiated squamous cell carcinoma. He was treated with two cycles of 5-fluorouracil and cisplatin and concomitant radiotherapy (6 MeV linear photon accelerator, total dose of 54 Gy), with complete remission. The control colonoscopy and endoscopy after six months of treatment were normal. Forty-five days before coming to the clinic again, he had a painful perianal and intergluteal erosion with circinate pustular borders and some isolated pustules, with surrounding erythema (Fig. 1). He was hospitalized and received systemic antibiotics (ceftriaxone and clindamycin) and famciclovir. A computed tomography on admission ruled out tumor recurrence, showing a normal anal canal. An incisional biopsy was performed, which showed pseudoepitheliomatous hyperplasia with microabscesses of inflammatory cells (neutrophils and eosinophils) (Fig. 2). In some microscopic fields, clefts with isolated keratinocytes next to microabscesses were observed (Fig. 3).

## What is your diagnosis?

a) Ulcerated herpes simplex associated with immunosuppression
b) Ulcerated radiodermatitis
c) Pemphigus vegetans
d) Tinea associated with immunosuppression

The histopathological findings were suggestive of pemphigus vegetans, which may present with acantholytic clefts associated with polymorphonuclear microabscesses,^1^ unlike classic pemphigus vulgaris in which the cleavage is accompanied by little inflammation.

**Figure 4:**
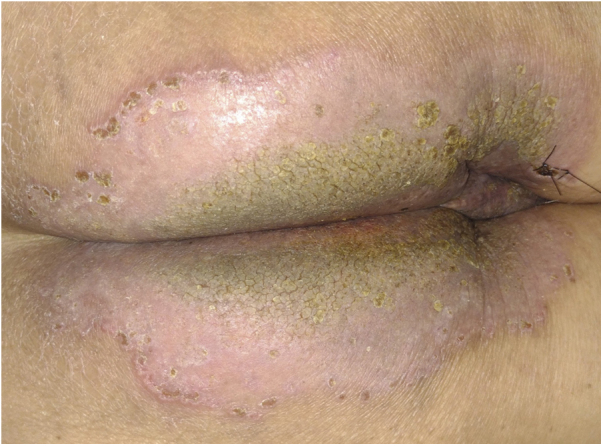
Clinical aspect with significant improvement after seven days of treatment, without the circinate pustular borders and mild verrucosity in the center.

There was no therapeutic response to systemic antibiotics and antivirals initiated before the results of the investigation, and there was significant regression with oral prednisone (40 mg) and topical betamethasone. After seven days, the pustular border disappeared, and there was total symptom regression, with the appearance of some hyperkeratotic and verrucous central areas (Fig. 4). Later, the result of indirect immunofluorescence was positive for IgG, with an intercellular pattern, 1:80 titer.

There are several reports of radiotherapy-induced pemphigus vulgaris, but not of the vegetans variant. Reported cases are restricted to the irradiated area,^2^, ^3^ (similarly to the case described here) or generalized.^4^, ^5^ There have also been reports of pemphigus foliaceus induction.^6^

The mechanism of induction of autoimmune acantholytic diseases by radiotherapy must be complex. One possibility is the antigenic alteration by radiation;^1^ another possibility would be the alteration of the immune response. Corroborating the hypothesis of antigenic alteration, there are reports of laboratory investigation with immunoblotting demonstrating antibodies against antigens other than desmogleins.^7^

This case demonstrates the possibility that pemphigus vegetans lesions may occur locally on a previously irradiated area.

## Financial support

None declared.

## Authors’ contributions

Hiram Larangeira de Almeida Jr.: Approval of the final version of the manuscript; design and planning of the study; drafting and editing of the manuscript; collection, analysis, and interpretation of data; intellectual participation in the propaedeutic and/or therapeutic conduct of the studied cases; critical review of the literature; critical review of the manuscript.

Antônia Larangeira de Almeida: Approval of the final version of the manuscript; design and planning of the study; drafting and editing of the manuscript; collection, analysis, and interpretation of data; intellectual participation in the propaedeutic and/or therapeutic conduct of the studied cases; critical review of the literature; critical review of the manuscript.

Pedro Henrique Evangelista Martinez: Approval of the final version of the manuscript; design and planning of the study; drafting and editing of the manuscript; collection, analysis, and interpretation of data; intellectual participation in propaedeutic and/or therapeutic conduct of the studied cases; critical review of the literature; critical review of the manuscript.

Ana Letícia Boff: Approval of the final version of the manuscript; design and planning of the study; drafting and editing of the manuscript; collection, analysis, and interpretation of data; critical review of the literature; critical review of the manuscript.

## Conflicts of interest

None declared.
